# Layered Foam/Film Polymer Nanocomposites with Highly Efficient EMI Shielding Properties and Ultralow Reflection

**DOI:** 10.1007/s40820-021-00759-4

**Published:** 2021-12-07

**Authors:** Li Ma, Mahdi Hamidinejad, Biao Zhao, Caiyun Liang, Chul B. Park

**Affiliations:** 1 Department of Mechanical and Industrial Engineering, University of Toronto, 5 King’s College Road, Toronto, ON M5S 3G8 Canada; 2 Institute for Manufacturing, Department of Engineering, University of Cambridge, Cambridge, CB3 0FS UK; 3 Laboratory of Advanced Materials, Department of Materials Science, Collaborative Innovation Center of Chemistry for Energy Materials, Fudan University, Shanghai, 200438 People’s Republic of China; 4 Henan Key Laboratory of Aeronautical Materials and Application Technology, School of Material Science and Engineering, Zhengzhou University of Aeronautics, Zhengzhou, Henan 450046 People’s Republic of China; 5 CAS Key Laboratory of High-Performance Synthetic Rubber and Its Composite Materials, Changchun Institute of Applied Chemistry, Chinese Academy of Sciences, Changchun, 130022 People’s Republic of China

**Keywords:** 2D MXene nanosheets, SiC nanowires, Layered foam/film polymer nanocomposites, Microcellular structure, Absorption-dominated EMI shielding

## Abstract

Lightweight, high-efficiency and low reflection electromagnetic interference (EMI) shielding polymer composites are greatly desired for addressing the challenge of ever-increasing electromagnetic pollution. Lightweight layered foam/film PVDF nanocomposites with efficient EMI shielding effectiveness and ultralow reflection power were fabricated by physical foaming. The unique layered foam/film structure was composed of PVDF/SiCnw/MXene (Ti_3_C_2_T_x_) composite foam as absorption layer and highly conductive PVDF/MWCNT/GnPs composite film as a reflection layer. The foam layer with numerous heterogeneous interfaces developed between the ﻿SiC nanowires (SiCnw) and 2D MXene nanosheets imparted superior EM wave attenuation capability. Furthermore, the microcellular structure effectively tuned the impedance matching and prolonged the wave propagating path by internal scattering and multiple reflections. Meanwhile, the highly conductive PVDF/MWCNT/GnPs composite (~ 220 S m^−1^) exhibited superior reflectivity (R) of 0.95. The tailored structure in the layered foam/film PVDF nanocomposite exhibited an EMI SE of 32.6 dB and a low reflection bandwidth of 4 GHz (R < 0.1) over the Ku-band (12.4 − 18.0 GHz) at a thickness of 1.95 mm. A peak SE_R_ of 3.1 × 10^–4^ dB was obtained which corresponds to only 0.0022% reflection efficiency. In consequence, this study introduces a feasible approach to develop lightweight, high-efficiency EMI shielding materials with ultralow reflection for emerging applications.

**Supplementary Information:**

The online version contains supplementary material available at 10.1007/s40820-021-00759-4.

## Highlights

- The successful fabrication of layered foam/film structure via the crystal melting temperature mismatch for two grades of PVDF resin in a batch foaming process.
- Developing the heterostructure interfaces between SiC nanowires and MXene (Ti_3_C_2_T_x_) nanosheets.
- Achieving efficient electromagnetic interference shielding effectiveness with ultralow reflectivity.

**Supplementary Information:**

The online version contains supplementary material available at 10.1007/s40820-021-00759-4.

## Graphical abstract

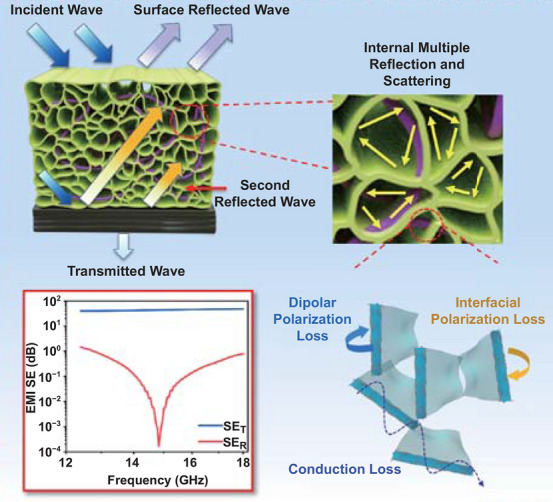

## Introduction

The rapid development of fifth-generation (5G) wireless systems, satellite communications and portable electronic devices is contributing to intensified electromagnetic wave emissions to the environment, which results in severe electromagnetic pollution [1–3]. The electromagnetic interference (EMI) can lead to malfunction of sensitive electronic components and system failure and may endanger human health [4–7]. To address the concerns caused by EMI pollution, substantial efforts have been devoted to developing high-efficiency EM wave attenuation and EMI shielding materials [8–12].

Efficient EMI shielding can be achieved by materials with high electrical conductivity [13]. For instance, 2D transition metal carbides (MXene) have recently been reported as promising EMI shielding materials owing to their superior electrical conductivity (~ 4500 S cm^−1^) [14]. Shahzad et al. [15] demonstrated an ultraefficient EMI shielding effectiveness (SE) of 92 dB for a freestanding film of Ti_3_C_2_T_x_ MXene at a thickness of 45 μm. Iqbal et al. [16] demonstrated that the shielding performance of freestanding MXene films could be further enhanced through heat treatment; for instance, heat-treated Ti_3_CNT_x_ MXene films offered an EMI SE of 116 dB at a thickness of 40 μm. Incorporating highly conductive nanomaterials such as MXene nanosheets in manufacturing polymer composites has shown great promise as efficient EMI shielding materials. Polymer composites have an attractive array of properties such as light weight, ease of processing, chemical stability, low cost and adjustable electrical and thermal properties [17–20]. The heterostructured interfaces, such as GnPs/SiC [21], MXene /Ni [22], SiC/C [23], NiCo@C/ZnO [24], C/MOS_2_ [25], Fe/MnO@C [26] and CNTs/Fe [27], constructed in the composite matrix can be a promising way to promote the EM wave dissipation capability by dielectric loss and/or magnetic loss and eventually convert the energy into heat.

In spite of the interesting properties of conductive polymer composites with outstanding EMI SE [28, 29], the severe impedance mismatch between solid and air results in significant reflection of the incident EM wave, leading to secondary EMI pollution in the environment. For instance, Song et al. [30] reported a highly conductive PDMS/cellulose carbon aerogel/rGO composite (75 S m^−1^) which exhibited an optimized EMI SE of 44 dB with SE_R_ of 7 dB. This means 99.996% of the incident EM waves are shielded, but 80% of waves are reflected at the surface. Therefore, absorption-dominated EMI shielding materials with high EM wave dissipation capability are in great demand [31–33].

Conductive polymer composite foams with microcellular structure can effectively tune the impedance matching and enhance the attenuation of EM waves via internal scattering and multiple reflections within the cellular structure [17, 19, 34–39]. Zhao et al. [17] demonstrated absorption-dominated EMI shielding properties for microcellular PVDF/10 wt% GnPs composite foams. Benefiting from the unique cellular structure, the composite foam exhibited an EMI SE of 27 dB with a void fraction (VF) of 48%, and the corresponding reflection effectiveness SE_R_ was only 2.6 dB, which indicated a reflection efficiency of 46% for the incident EM wave. To develop shielding materials with ultralow reflection, the surface impedance matching should be carefully tuned by increasing the degree of foaming and decreasing the effective dielectric permittivity [40, 41]. However, such materials can hardly obtain efficient EMI SE due to insufficient electrical conductivity and dissipation capability [42–44]. Zhao et al. [42] demonstrated an optimized SE_R_ of 1.6 dB but insufficient EMI SE of 12.8 dB by increasing the VF of composite foam from 36 to 60%. It is still challenging to fabricate efficient EMI shielding material with ultralow reflectivity. To address this drawback, Duan et al. [45] introduced a graded conductive polymer composite foam via freeze drying. Such gradient structures can achieve both low reflectivity and high EMI SE by combining (i) the absorption layer, with excellent impedance matching and high attenuation capability, and (ii) the reflection layer with superior electrical conductivity and high reflection capability. A waterborne polyurethane/FeCo@rGO/Ag-coated expanded polymer bead (EBAg) composite foam exhibited an average EMI SE of 84 dB with an average SE_R_ of 0.32 dB [46, 47]. Despite the importance of shielding materials with ultralow reflection, the field is at an early stage, and there are very few research works devoted to the manufacture of efficient ultralow reflection shielding materials.

In this work, a novel design of lightweight, layered foam/film PVDF nanocomposite with efficient EMI shielding effectiveness and ultralow reflection characteristics is reported. The foam/film composites were fabricated based on the mismatched crystal melting temperatures of two grades of PVDF resin from the batch foaming process. The layered foam/film nanocomposites were composed of an upper PVDF/SiCnw@MXene composite foam as impedance matching and wave attenuation layer, and a bottom highly conductive PVDF/MWCNT/Graphene nanoplatelets (GnPs) composite film as the reflection layer. A percolated MWCNT/GnPs network constructed in the PVDF matrix resulted in an efficient EMI SE (33.49 dB) with high reflectivity due to superior electrical conductivity (~ 220 S m^−1^) and high impedance mismatch. The numerous heterogeneous interfaces developed between SiCnw and MXene nanosheets in the PVDF matrix enhanced the EM wave attenuation capability by interfacial polarization loss and conduction loss. Meanwhile, the incorporation of microcellular structure can effectively tune the surface impedance matching and internal scattering. The degree of foaming and composition of SiCnw/MXene hybrids were optimized to maximize low reflection bandwidth (reflection less than 10%) and minimize the reflection efficiency over the Ku-band (12.4 − 18.0 GHz). As a result, this study introduces a simple method to develop lightweight, efficient low reflection EMI shielding materials.

## Materials and Methods

### Materials

Commercial grade polyvinylidene fluoride-co-hexafluoropropylene (PVDF-HFP), Kynar2800, and polyvinylidene fluoride (PVDF), Kynar740, with specific gravity of 1.78 and 1.8 g cm^−3^, respectively, were kindly donated by ArkemaNA. SiC nanowires (SiCnw), with a diameter of 50–600 nm, were purchased from Nanjing XFNano Materials Tech Co., Ltd. Graphene nanoplatelet (GnP) powder was purchased from SuperiC Technology Ltd. (China). Carbon nanotube (CNT) powder, NC7000t, was obtained from Nanocyl SA, Belgium. Hydrochloric acid ACS reagent Grade (12 Normal) was purchased from BioShop Canada Inc. Lithium fluoride (LiF) powder (≥ 99.9%), N, N-dimethylformamide (DMF) and poly (diallyl dimethylammonium chloride) (PDDA) were purchased from Sigma-Aldrich. ${\text{Ti}}_{3} {\text{AlC}}_{2}$ powder (400 mesh) was purchased from 11 Technology Co., Ltd. (China).

### Preparation of PVDF-HFP/SiCnw@MXene Nanocomposites

**Fig. 1:**
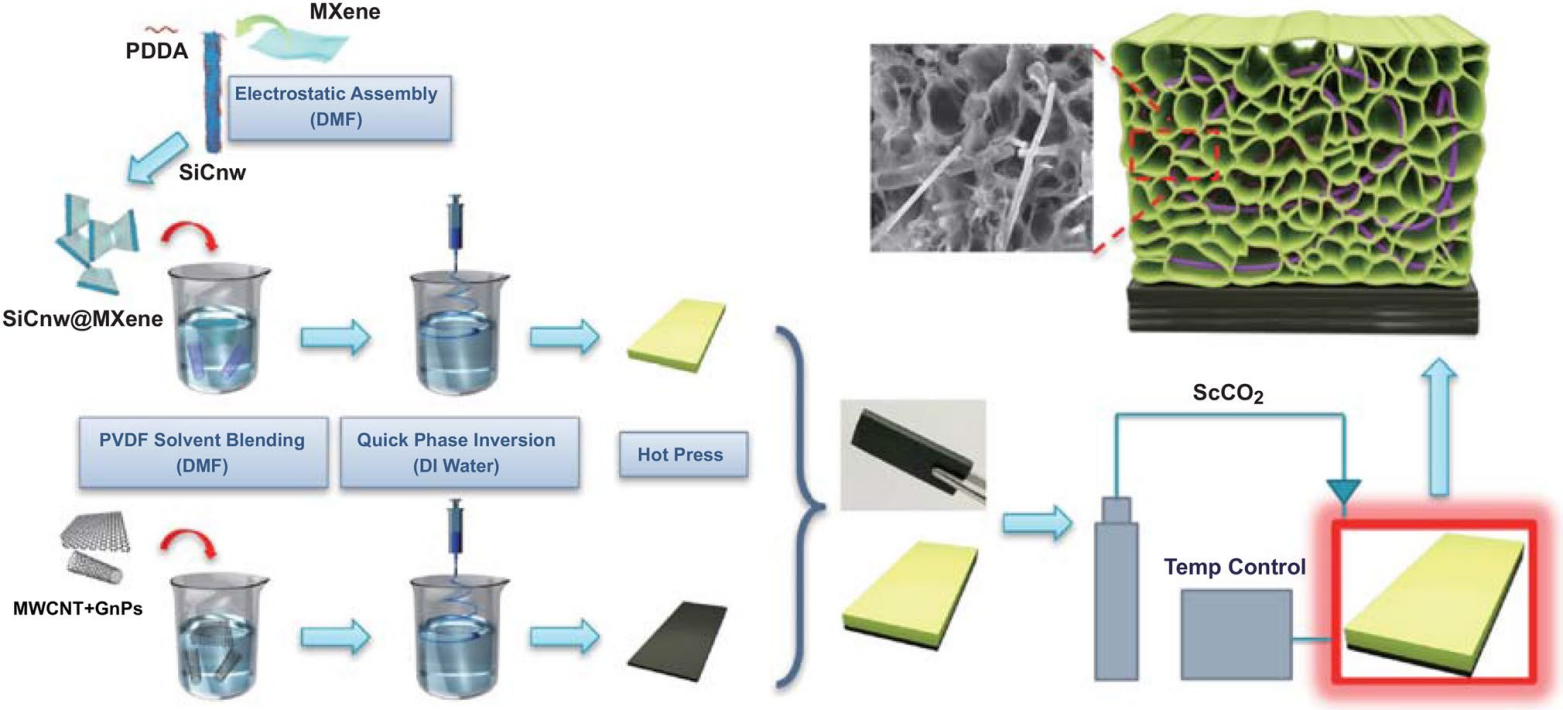
Schematic illustration of layered foam/film PVDF nanocomposite fabrication

The synthesis process of few-layer Ti_3_C_2_T_x_ MXene and modified SiCnw was introduced in previous work, and more details can be seen in ESI [48]. The schematic illustration of fabrication is shown in Fig. 1. Firstly, 0.07 g of Ti_3_C_2_T_x_ MXene was dispersed in 30 mL of DMF followed by 15 min sonication in an Ar environment. Then, 0.49 g of the modified SiCnw was added to the MXene/DMF solution and sonicated for another 15 min. The SiCnw/MXene was then uniformly dispersed in DMF solution under continuous stirring for 30 min. The modified SiCnw and MXene 2D sheets can be assembled together, driven by electrostatic force during the process. After that, 1.2 g of PVDF-HFP was dissolved and dispersed in the mixture via continuous stirring for 3 h at 70 °C. The resulting PVDF-HFP/SiCnw@MXene/DMF solution was then injected into a water tank with magnetic stirring at the bottom via a syringe for quick phase inversion. Finally, after drying in a vacuum oven at 70 °C overnight, the product was hot-pressed at 200 °C to obtain PVDF/30 wt% SiCnw@MXene nanocomposites (1.48 × 15.8 × 20 mm^3^). Different compositions of SiCnw and MXene hybrids were applied: SiCnw@MXene 5:1 (0.467 and 0.093 g), SiCnw@MXene 7:1 (0.49 and 0.07 g), SiCnw@MXene 9:1 (0.504 and 0.056 g) and neat SiCnw (0.56 and 0 g).

### Preparation of PVDF/GnPs/MWCNT Nanocomposites

Firstly, 0.1 g of GnPs and 0.1 g of MWCNTs were dispersed in 25 mL of DMF and sonicated for 30 min. Then, 0.8 g of PVDF was dissolved and dispersed in the solution via continuous stirring for 3 h at 70 °C. After mixing, the same quick phase inversion technique was applied. Finally, the product was hot-pressed at 200 °C to obtain PVDF/10 wt% GnPs/10 wt% MWCNT nanocomposite (0.4 × 15.8 × 20 mm^3^).

### Fabrication of Layered Foam/Film PVDF Nanocomposites

As shown in Fig. 1, 1.8 mm of PVDF/SiCnw@MXene composite and 0.4 mm of PVDF/GnPs/MWCNT composite were layered and placed in a mold (2.2 × 15.8 × 20 mm^3^), and the two composites were hot-pressed together at 200 °C. Then, the double layer composites obtained were placed in a high-pressure batch foaming chamber, filled with 2000 psi supercritical carbon dioxide, and saturated at various temperatures for 30 min. The saturation temperatures were set below the onset temperature of PVDF crystal melting (163 °C) to avoid foaming the film layer, while the PVDF-HFP layer could still be foamed. After saturation, the gas was quickly released and the chamber quenched in an ice bath to obtain the layered foam/film PVDF nanocomposites, where PVDF-HFP/SiCnw@MXene foam sits on PVDF/GnPs/MWCNT film. The influence of saturation temperature (128 to 143 °C) on the VF of foamed layer was investigated, as shown in Table S1. In this study, PVDF-HFP/SiCnw@MXene foams with VF around 45%, 55% and 65% were selected to study the effect of VF on their EMI shielding performance.

### Characterization

The samples’ broadband alternating current (AC) conductivity was tested by a KEYSIGHT impedance analyzer (E4990A, 20 Hz to 10 MHz). Field emission scanning electron microscopy (SEM, FEI Quanta FEG 250, with EDX) and transmission electron microscopy (TEM, Tecani G2) were applied to investigate and observe microstructure and morphology of the samples. An X-ray diffractometer (XRD, Rigaku MiniFlex 600) using Cu-Ka as a radiation resource was applied to investigate the crystal structure of samples. The elemental chemical bonding in MXene was confirmed by X-ray photoelectron spectroscopy (XPS, Thermo Scientific Theta Probe) using Al-Ka as a radiation resource. The temperature-dependent storage modulus of the samples was investigated by the Dynamic mechanical analysis (DMA) instrument (TA DMA q800, tensile fixture, 30–80 °C, oscillation frequency 1 Hz, oscillation strain 0.02%).

### Measurement of Electromagnetic Wave Interference Shielding Effectiveness

The S parameters (S_11_ and S_21_) of each sample were collected by using the waveguide mode in the frequency range 12.4−18.0 GHz (Ku-band) on a vector network analyzer (VNA, KEYSIGHT, PNA-L Series N5234B). Samples were cut and trimmed into a 15.8 × 7.9 mm^2^ rectangular shape to precisely fit the waveguide fixture. The reflection shielding effectiveness (SE_R_) and the total shielding effectiveness (SE_T_) can be determined by the following equations using S_11_ and S_21_ parameters [21, 37]:

1 $$R = \left| {S_{11}^{2} } \right|,T = \left| {S_{21}^{2} } \right|, \, A + T + R = 1$$

2 $${\text{SE}}_{{\text{R}}} = - 10{\text{log}}\left( {1 - {\text{R}}} \right)$$

3 $${\text{SE}}_{{\text{T}}} = - 10{\text{log}}\left( {\text{T}} \right)$$

where A, T and R are the absorptivity, transmissivity and reflectivity for the incident EM wave, respectively. Moreover, the absorption effectiveness of the incident wave can be described as following [17, 49]:

4 $${\text{SE}}_{A} = - 10\log \left( {1 - \frac{A}{1 - R}} \right) = {\text{SE}}_{T} - {\text{SE}}_{R} - {\text{SE}}_{M}$$

when the SE_*T*_ is larger than 15 dB, multiple reflections (SE_M_) are generally negligible. The influence of sample thickness on EMI shielding performance was investigated by trimming the sample thickness using sandpaper.

## Results and Discussion

### Microstructure and Morphology

**Fig. 2:**
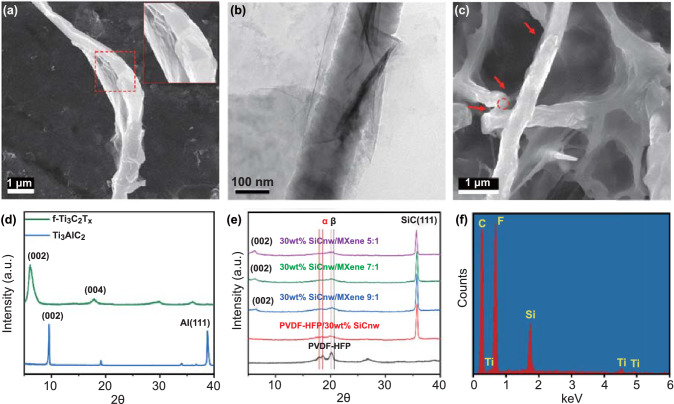
**a** SEM image of self-assembled SiCnw@Ti_3_C_2_T_x_ nanostructure; **b** TEM image of SiCnw@f- Ti_3_C_2_T_x_; **c** EDX-SEM image of PVDF-HFP/SiCnw@MXene composite foam (30 wt%, 45% VF); **d** XRD patterns of Ti_3_AlC_2_, Ti_3_C_2_T_x_; **e** XRD patterns of PVDF-HFP composites; **f** EDX pattern of the red circle in **c**

Driven by the electrostatic force, the flexible 2D Ti_3_C_2_T_x_ nanosheets can wrap over the surface of modified 1D SiCnw [48, 50], and the microstructure and morphology of such a heterostructure are illustrated in Figs. 2 and S1. The SiCnw@MXene heterostructure can also be observed in PVDF /SiCnw@MXene composite foam. As shown in Figs. 2c, f and S1, the EDX-SEM image confirms that the SiCnw@MXene heterostructure is preserved after solvent casting, hot pressing and foaming.

The phase and crystal structure of as-prepared MXene film and fabricated nanocomposites were examined using XRD analysis. Also, after selective etching, the peak corresponding to the (002) crystal planes of Ti_3_AlC_2_ shifted from ~ 9.7° to ~ 6.6° because of an increase in the d-spacing of the Ti_3_C_2_T_x_ (Fig. 2d) [15, 51, 52]. The absence of the (111) plane peak of Al in X-Ray spectra of the few-layer Ti_3_C_2_T_x_ nanosheets is an indication of successful manufacture of MXene nanosheets. The XRD pattern of PVDF/SiCnw@MXene composites with various filler compositions is shown in Fig. 2e; the peak at ~ 35.7° corresponds to the (111) plane of SiC [23, 53, 54]. The intensity of the (002) plane of MXene increases with MXene content in the composite. Also, the XRD pattern of the PVDF/SiCnw@MXene composites illustrates that the incorporation of Ti_3_C_2_T_x_ nanosheets and SiCnw could promote the formation of PVDF β crystals. The promoted β phase can be attributed to the extensional stress around the embedded filler in the PVDF melt [55], which can help to align polymer chains and to form the electroactive β phase crystal [56].

Electronic states of the Ti element within Ti_3_C_2_T_x_ MXene nanosheets can be found in Fig. S2. The fabrication of PVDF/MWCNT/GnPs nanocomposite is supported by the XRD spectra in Fig. S3. The characteristic peak of MWCNT and GnPs was detected around 26°.

**Fig. 3:**
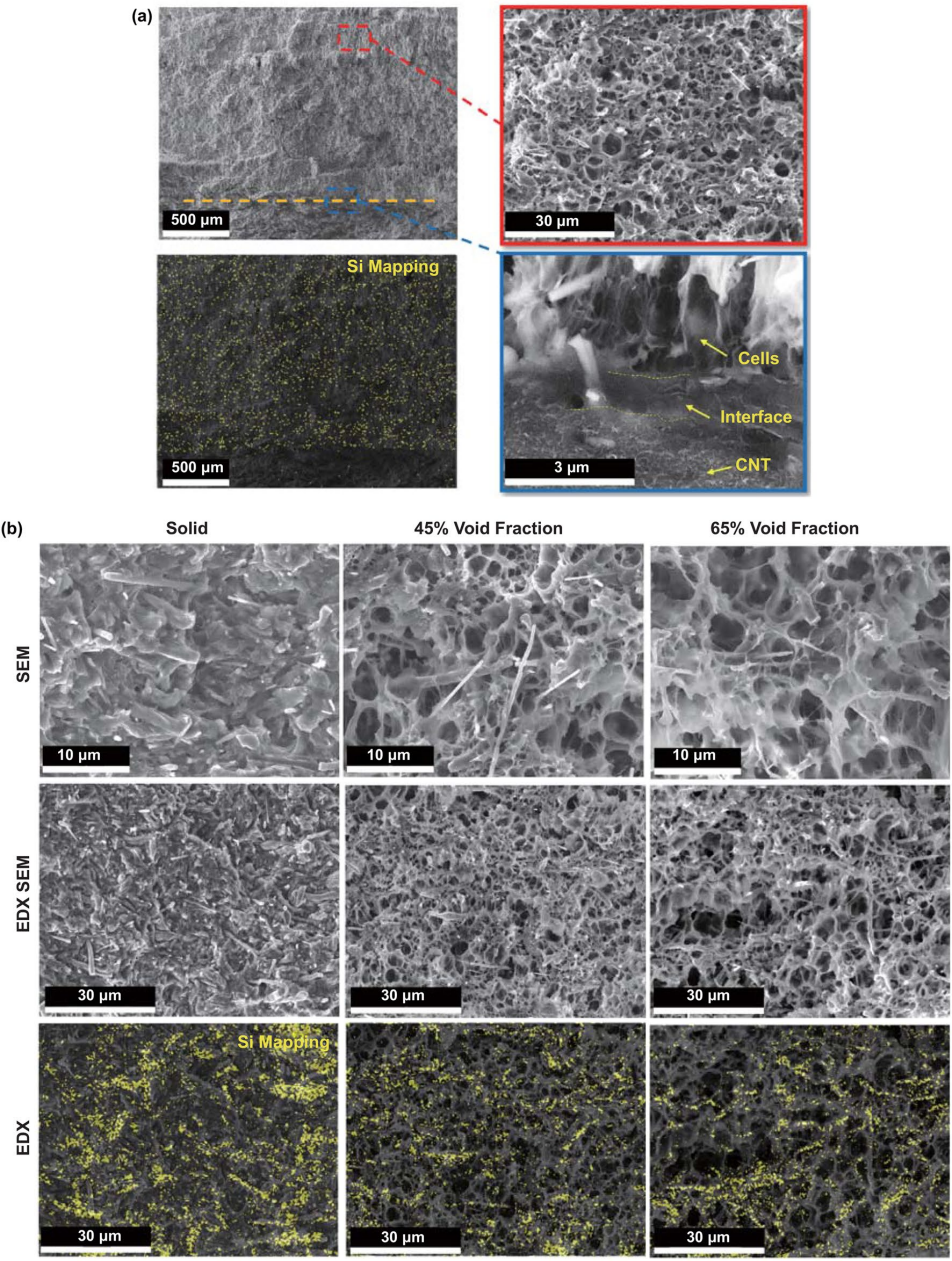
Cross-sectional SEM images and Si EDX mapping of **a** the layered foam/film PVDF nanocomposite (upper layer VF: 45%); **b** upper foamed layer with a different void fraction (30%SiCnw@MXene7:1, VF: 0%, 45% and 65%)

Figure 3a shows the cross-sectional SEM images of layered foam/film PVDF nanocomposites (PVDF/SiCnw@MXene composite foam and PVDF/MWCNT/GnPs composite film). The microstructure of the layered foam/film PVDF nanocomposites before foaming is shown in Fig. S4d, e. The microcellular morphology in Fig. 3a confirms the successful foaming of the upper PVDF composite. An appropriate foaming temperature window (128 to 162 °C) of the PVDF composites guarantees the formation of microcellular structure in the upper layer and keeping the bottom layer solid, as shown in Figs. 3a and S4a, b. The endothermal plots for two grades of PVDF are presented in Fig. S5. Therefore, the existing crystal lamellae in the PVDF matrix will be the gas barrier and prevent gas molecules getting into the bottom high melting temperature PVDF matrix [57]. It is worth noting that foaming of the upper layer composite is constrained around the interface, and only some small cells are observed. In the meantime, a thin layer of interface stayed in solid state between two layers, which can be the indication of blending of two grades of PVDF at the interface during the hot pressing. The interface layer is visible in the solid PVDF nanocomposite, as shown in Fig. S4f.

The high magnification SEM images of upper foamed absorbers with different VF (0%, ~ 45%, and ~ 65%) are shown in Fig. 3b. In the case of the samples before foaming (solid), as shown in the first column of Fig. 3b, a percolated and agglomerated SiCnw/MXene network was constructed in the upper layer via solvent casting (PVDF/SiCnw@MXene (30 wt%, 7:1)). As shown in the second and third columns of Fig. 3b, by inducing foaming, the cells nucleate and form the interface between the filler and polymer matrix.

According to the heterogeneous nucleation theory, driven by the thermal instability of the saturated gas molecules in the molten polymer after pressure drop, nucleation of cells will be favored at the interface between the filler and polymer matrix due to reduction in activation Gibbs free energy [39, 55, 58]. Also, the filler particles could be reoriented and realigned around the growing bubble [38, 43, 59].

It can be seen in the EDX mapping of Si element (Fig. 3b) that cells are surrounded by a percolative and distributed SiCnw network. The increase in the VF from ~ 45% to ~ 65%, as shown in the second row of Fig. 3b, increased the average cell size from 3.08 to 4.79 μm. Figure 3 illustrates that surrounding SiCnw/MXene networks were separated as the bubble grew.

The Dynamic mechanical analysis (DMA) presented in Fig. S6 confirms that the fabricated foam/film PVDF nanocomposites offer excellent stiffness as compared to the neat PVDF. As shown in Fig. S6, the incorporation of nanofillers in the PVDF resulted in a significant stiffening effect in the temperature range of 30–80 °C. The storage modulus of 1.5 mm layered PVDF nanocomposite (30 wt% SiCnw@MXene7:1) increased from 603 to 3,901 MPa at 30 °C, as compared to neat PVDF with the same thickness. By inducing 45% and 65% void fraction into the upper absorption layer, the storage modulus of layered foam/film PVDF nanocomposites decreased to 1,885 and 1,026 MPa, respectively.

### Electrical Properties and Electromagnetic Wave Interference Shielding Performance of the Nanocomposites

In general, the EMI shielding and impedance matching properties of EMI shielding materials are governed by electrical conductivity. Increasing the electrical conductivity can enhance the EMI SE and EM wave dissipation via conduction loss [21, 50]. However, excessively high conductivity can result in high reflectivity because of impedance mismatch [60].

To better understand the underlying mechanisms of the layered foam/film PVDF nanocomposites, the electrical and EMI performance of each layer was investigated separately. The AC conductivity for each PVDF nanocomposite with various filler compositions is shown in Fig. S7. Incorporation of MXene nanosheets in PVDF/30 wt% SiCnw increased AC conductivity at 100 Hz from 4.1 × 10^–7^ to 3.5 × 10^–6^ S m^−1^. When the mass ratio of SiCnw to MXene decreased from 9:1 to 5:1, the addition of MXene increased the conductivity of the nanocomposites by more than two orders of magnitude at 100 Hz. The higher conductivity of the matrix could increase the absorption of EM waves by conduction loss. On the other hand, the bottom reflection layer (PVDF/10 wt% MWCNT/10 wt% GnPs) exhibits frequency-independent behavior, which is an indication of a well-developed percolated conductive network in the matrix, with an AC conductivity of 220 S m^−1^ at 100 Hz. The high conductivity could provide a significant impedance mismatch and lead to surface reflection of EM waves.

**Fig. 4:**
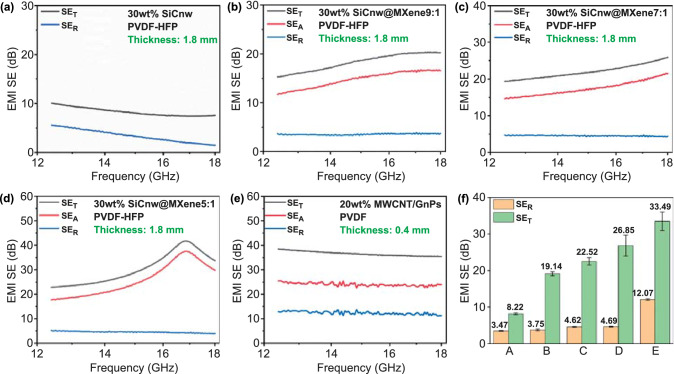
**a-d** EMI shielding performance of PVDF/30 wt% SiCnw@MXene nanocomposites with various filler compositions; **e** EMI shielding performance of PVDF/10 wt% MWCNT/10 wt% GnPs nanocomposite; **f** Average SE_R_ and SE_T_ of PVDF nanocomposites from **a-e**

**Fig. 5:**
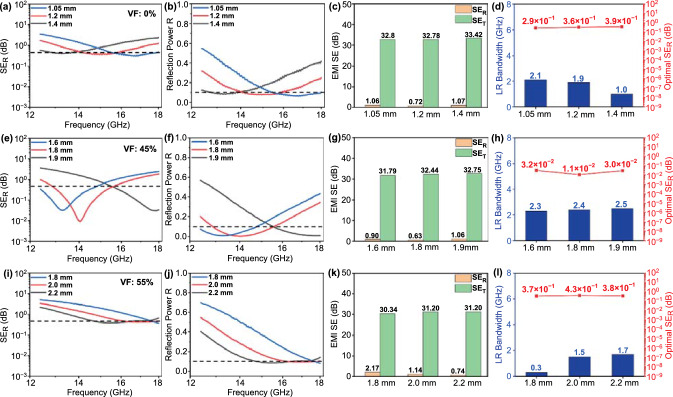
EMI shielding performance for layered foam/film PVDF nanocomposites (30 wt% SiCnw) corresponding to different matching thicknesses of absorption layer; **a, e, i** SE_R_; **b, f, j** reflectivity R; **c, g, k** average value of SE_R_ and SE_T_; **d, h, l** low reflection bandwidth and minimal SE_R_ value. Dependence of the VF: **a-d** 0% VF; **e–h** 45% VF; and **i-l** 55% VF

Dielectric loss (including dipolar and interfacial polarization loss) is another key factor governing the EM wave dissipation capability [61, 62]. According to the Maxwell–Wagner–Sillars (MWS) theory [63, 64], the development of unique heterostructures of SiCnw/MXene hybrid in the PVDF matrix can induce significant interfacial polarization loss by initiating accumulation of charge at the heterogeneous interfaces [65–67]. The EMI shielding properties of the individual layer of solid PVDF nanocomposites over the Ku-band (12.4–18 GHz) with various filler compositions are presented in Fig. 4. Incorporation of MXene nanosheets in the PVDF/30 wt% SiCnw, slightly increased the average SE_R_ from 3.47 to 3.75 dB for the PVDF/SiCnw@MXene 9:1 composite, while the average SE_T_ was significantly promoted from 8.22 to 19.14 dB. The reflection effectiveness was almost unchanged. Thus, the enhanced EMI shielding performance is mainly attributed to the enhanced absorptivity (A). Such enhancement indicates that the incorporation of SiCnw@MXene heterostructures could significantly promote the absorption of EM waves by interfacial polarization loss [48, 68]. Meanwhile, the resonance shielding peak obtained in Fig. 5d can be attributed to the multi-reflection and scattering of propagating EM wave among the dispersed SiCnw@MXene within the matrix, which results in the enhanced absorption of incident EM wave via dielectric loss [69, 70].

Moreover, the formation of β crystals in the PVDF matrix can help to enhance the dielectric constant by inducing dipolar polarization [71–73]. Then, based on the MWS theory, an increased dielectric constant can further enhance the interfacial polarization. Under alternating EM fields, both dipolar and interfacial polarization can turn into dielectric loss at high frequency due to relaxation processes, which then attenuate the incident EM wave. Furthermore, both SE_R_ and SE_T_ of PVDF/SiCnw@MXene composites gradually increased with increase in MXene content.

In previous studies, GnPs and MWCNT exhibited an excellent synergistic effect in PVDF nanocomposites, which resulted in enhanced conductivity and EMI shielding effectiveness [28]. As shown in Fig. 4e, f, the 0.4 mm thick reflection layer (PVDF/10 wt% MWCNT/10 wt% GnPs) had an average EMI SE of 33.49 dB and average SE_R_ of 12.07 dB. This indicates that more than 99.95% of the incident EM wave was shielded and 95% of the incident EM wave was reflected from the surface of the composite film.

As mentioned in the previous sections, the layered foam/film PVDF nanocomposites were composed of PVDF/SiCnw@MXene composite foam as an absorption layer and highly conductive PVDF/MWCNT/GnPs composite film as a reflection layer. The incident EM wave can penetrate and propagate from the upper absorption layer. To optimize the filler composition of SiCnw/MXene in the PVDF matrix as the absorber, mass ratios of SiCnw and MXene of 1:0, 9:1, 7:1, and 5:1 were investigated. The EMI SE of the layered foam/film PVDF nanocomposites (30 wt% SiCnw) over the 12.4–18 GHz (Ku-band) with various VFs is presented in Fig. S8. Due to the excellent shielding performance of the reflection layer (0.4 mm PVDF/10 wt% MWCNT/10 wt% GnPs), all nanocomposites exhibited an average SE_T_ above 30 dB, regardless of the thickness of the absorption layer, which means over 99.9% of incident EM wave will be shielded. Meanwhile, from Fig. 5, all the 30 wt% SiCnw layered PVDF nanocomposites exhibited a minimum value of SE_R_ over the Ku-band. This minimum value of SE_R_ shifts toward higher frequency with decreasing thickness of the absorption layer. This can be explained by the law of quarter wavelength cancelation [74, 75]:

5 $$t_{m} = n\frac{\lambda }{4} = n\frac{c}{{4f_{m} \sqrt {\left| {\mu_{r} } \right|\left| {\varepsilon_{r} } \right|} }} \left( {n = 1, 3, 5 \ldots } \right)$$

where $t_{m}$ is the thickness of the absorption layer, ${\uplambda }$ is the wavelength of EM wave, $f_{m}$ is the peak frequency with the minimal SE_R_, $\left| {\mu_{r} } \right| and \left| {\varepsilon_{r} } \right|$ refer to the complex permeability and permittivity of the sample, and c is the speed of the light. When the thickness of the absorption layer equals a quarter of the penetrating wavelength multiplied by an odd number, the EM wave will be dissipated by interference, where the second reflection of the wave has 180° phase difference relative to the surface reflection. It is worth noting that, in EM wave attenuation-related applications, the bandwidth with more than 90% absorption efficiency is defined as the effective absorption band [76, 77]. Therefore, in this work, with an average shielding efficiency of 99.95%, the bandwidth with SE_R_ less than 0.45 dB will be defined as the low reflection bandwidth (LR bandwidth). SE_R_ less than 0.45 dB corresponds to a reflection efficiency of less than 9.85% (R < 0.1), in other words, over 90% of the incident EM waves will be absorbed. From Fig. 5d, the layered PVDF nanocomposites (30 wt% SiCnw, 0% VF) exhibited an optimal low reflection bandwidth of 2.1 GHz, and the corresponding peak SE_R_ obtained is 0.29 dB at an absorption layer thickness of 1.05 mm. Such absorption performance is attributed to the excellent dipolar polarization loss of the incorporated SiCnw [35].

By inducing foaming in the absorption layer with a 45% VF, the layered foam/film PVDF nanocomposites exhibited enhanced EM wave absorption performance, as shown in Fig. 5e-h. At a foam thickness of 1.8 mm, the layered foam/film PVDF nanocomposite exhibited a broader optimal LR bandwidth from 2.1 to 2.4 GHz and smaller corresponding minimal SE_R_ from 0.36 to 0.011 dB compared to its solid counterpart. This value corresponds to only 0.25% reflection efficiency. The presence of cellular structure can help tailor the impedance match by reducing the real permittivity of the absorber, and fewer incident EM waves will be reflected at the surface [35, 41]. Meanwhile, the microstructures shown in Fig. 4 can provide abundant solid–air interfaces where the propagating EM wave undergoes internal scattering within each bubble [19, 34, 41, 78]. This prolongs the propagation path of EM waves in the foam. Eventually, the propagating EM wave will be further attenuated in the foamed absorption layer.

The absorption phase exhibited a severe impedance mismatch by further increasing the void content from 45 to 55%. From Fig. 5i-l, the layered foam/film nanocomposite had a narrower optimal low reflection bandwidth of 1.7 GHz with an optimal SE_R_ of 0.38 dB at a foam thickness of 2.2 mm. In this case, the poor impedance mismatch is attributed to insufficient absorption region and dissipation capability of the over-foamed absorber. This is also evident in Fig. S9a, where the AC conductivity of the PVDF/30 wt% SiCnw composite foam (65% VF) is reduced by nearly 1 order of magnitude at 1 kHz, from 1.9 × 10^–7^ to 5.6 × 10^–8^ S m^−1^, as compared to the layered foam/film nanocomposite with 45% VF.

**Fig. 6:**
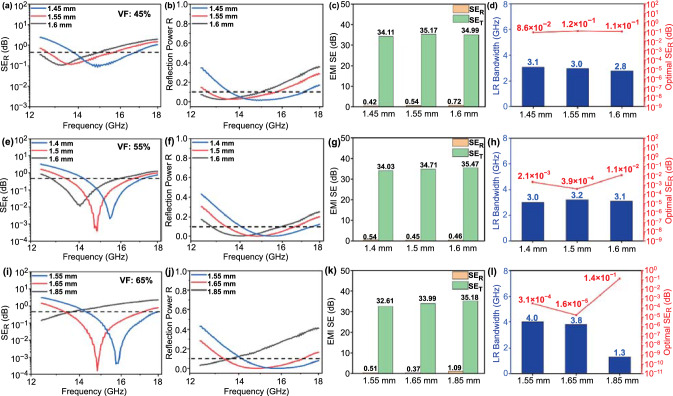
EMI shielding performance of layered foam/film PVDF nanocomposites (30 wt% SiCnw@MXene7:1) corresponds to different matching thicknesses of absorption layer; **a, e, i** SE_R_; **b, f, j** reflection power R; **c, g, k** average value of SE_R_ and SE_T_; **d, h, l** low reflection bandwidth and minimal SE_R_ value. Dependence of the VF: **a–d** 45% VF; **e–h** 55% VF; and **i–l** 65% VF

By incorporating MXene nanosheets in the absorption layer, the layered foam/film PVDF nanocomposites (30 wt% SiCnw@MXene7:1) exhibited enhanced EM wave attenuation capability, as shown in Figs. 6 and S10. With the same 45% VF (Fig. 6a-d), the layered foam/film PVDF nanocomposite (30 wt% SiCnw@MXene7:1) exhibited a broader optimal LR bandwidth of 3.1 GHz with a peak SE_R_ of 0.086 dB at a foam thickness of 1.45 mm, as compared to the layered foam/film PVDF nanocomposites without MXene nanosheets incorporated (30 wt% SiCnw). In this case, the broader low reflection bandwidth suggests that the heterostructures of SiCnw/MXene in the PVDF matrix can effectively tune the dissipation capability and impedance matching. Also, the foam thickness to achieve optimal low reflection bandwidth was reduced from 1.8 to 1.45 mm. This indicates enhanced dielectric permittivity of the absorber, attributed to the intensified interfacial polarization because of the addition of MXene nanosheets. The conduction loss of the layered foam/film PVDF nanocomposites is also enhanced with incorporation of MXene nanosheets. As shown in Fig. S9a, c, two absorbers (30 wt% SiCnw@MXene7:1 and 30 wt% SiCnw, 65% VF) had AC conductivity of 9.7 × 10^–7^ and 1.6 × 10^–7^ S m^−1^, respectively. The bottom reflection layer was removed by sanding when the ac conductivity of the upper absorption layer was measured.

By increasing the VF of absorber from 45 to 55% (Fig. 6e-h), the increased void content led to promoted impedance matching, where layered foam/film PVDF nanocomposite had a broader optimal low reflection bandwidth of 3.2 GHz, with a peak SE_R_ of 3.9 × 10^–4^ dB at a foam thickness of 1.5 mm. Moreover, by further increasing the VF of absorber to 65% (Fig. 6i-l), the layered foam/film nanocomposite had a broader LR bandwidth of 4.0 GHz with an optimal SE_R_ of 3.1 × 10^–4^ dB at a foam thickness of 1.55 mm. This SE_R_ value corresponds to only 0.0022% reflection efficiency. The density of the product should also be considered to evaluate the EMI shielding properties. At the optimal condition, SiCnw@MXene7:1 layered foam/film PVDF nanocomposite (65% VF) had a 42% of reduction in area density from 0.322 to 0.187 g cm^−2^, as compared to its original (30 wt% SiCnw) solid counterpart (Table 1).

**Table 1:** EMI shielding properties for layered foam/film PVDF nanocomposites with various filler compositions, VF and absorption layer thickness

Absorption layer filler content	Void fraction%	Total thickness (mm)	Area density g ${\text{ cm}}^{ - 2}$	Minimal ${\text{SE}}_{R}$ (dB)	Average ${\text{SE}}_{R}$ (dB)	Bandwidth ${\text{SE}}_{R}$ < 0.45 dB (GHz)
30 wt% SiCnw	0	1.6	0.322	0.36	0.72	1.9
	45	2.2	0.284	$1.11 \times 10^{ - 2}$	0.63	2.4
	55	2.6	0.281	0.38	0.74	1.4
30 wt% SiCnw@MXene 9:1	45	1.9	0.238	$1.48 \times 10^{ - 2}$	0.46	3.2
	55	2.4	0.259	$2.48 \times 10^{ - 6}$	0.58	3.0
	55	2.2	0.241	$5.41 \times 10^{ - 4}$	0.39	3.2
	55	2.0	0.223	$2.08 \times 10^{ - 4}$	0.63	3.5
	65	2.4	0.219	$5.41 \times 10^{ - 2}$	0.43	3.9
30 wt% SiCnw@MXene 7:1	45	1.85	0.239	$8.61 \times 10^{ - 2}$	0.42	3.1
	55	1.9	0.212	$3.90 \times 10^{ - 4}$	0.45	3.2
	65	2.05	0.194	$1.65 \times 10^{ - 4}$	0.37	3.8
	65	1.95	0.187	$3.10 \times 10^{ - 4}$	0.51	4.0

Reflection Layer **0.4 mm** (PVDF/20 wt% GnP@CNT)-Average $SE_{T}$ 33.49 dB

Increasing the VF can effectively tune the impedance matching and intensify internal scattering and multi-reflection, leading to a broader optimal low reflection bandwidth. However, excessively high void content can further break the embedded fillers apart around growing cells, leading to reduction in effective permittivity, conductivity and dissipation capability. The effect of the VF on the EMI shielding properties has been further explored in Fig. S11. The EM wave reflection performance for layered foam/film PVDF nanocomposites (30 wt% SiCnw@MXene9:1) with various VFs of the absorption layer at the same thickness has been investigated.

Moreover, with a higher MXene content, higher values of optimum VF can be achieved before compromising the absorption and dissipation capability. At a fixed VF of 55%, by increasing the MXene content (30 wt%, SiCnw:MXene from 1:0 to 9:1 and to 7:1), the SE_A_ of composite foams increased from 2.6 to 3.75 dB, and to 4.01 dB, respectively (Fig. S12a).

The summary of the experimental results presented in Table 1, the layered foam/film PVDF nanocomposite (30 wt% SiCnw@MXene9:1) had an optimal low reflection bandwidth broadened from 3.2 to 3.9 dB as the VF increased from 45 to 65%. The SiCnw@MXene9:1 layered foam/film PVDF nanocomposite (65% VF) showed a similar optimal low reflection bandwidth (~ 4 GHz) to those of the SiCnw@MXene7:1 layered foam/film nanocomposite (65% VF). However, the SiCnw@MXene9:1 layered foam/film PVDF nanocomposite (65%VF) had a higher matching thickness of 2.0 mm for optimal low reflection bandwidth. Also, the minimum SE_R_ obtained is two orders of magnitude higher than that of the SiCnw@MXene7:1 layered foam/film PVDF nanocomposite (65% VF). It is clear that the effective permittivity and dissipation capability of the SiCnw@MXene9:1 layered foam/film PVDF nanocomposite (65% VF) are insufficient due to over-foaming, as compared to the SiCnw@MXene7:1 layered foam/film nanocomposite (65% VF) (shown in Figs. S13 and S14). The EMI shielding properties of the absorption layers (without the reflection layer) are shown in Fig. S12a. The larger void fraction in absorption layers often leads to a lowered SE_R_. For instance, PVDF SiCnw@MXene foam (30 wt% 9:1, 55% VF, 1.8 mm) obtained a SE_R_ of 2.79 dB, while PVDF SiCnw@MXene foam (30 wt% 7:1, 65% VF, 1.8 mm) exhibited a SE_R_ of 2.39 dB regardless of the increased MXene content. In the meantime, both composite foams obtained a SE_A_ of around 3.75 dB, indicating that foam (7:1, 65% VF) obtained better impedance matching (lower reflectivity) while maintaining the high EM wave attenuation capability. Moreover, by increasing the MXene content (9:1 to 7:1), the AC conductivity of the absorption layer increased from 2.2 × 10^–7^ to 4.5 × 10^–7^ S m^−1^ at 1 kHz (Fig. S9b, c). Detailed EMI shielding properties of layered foam/film PVDF nanocomposite (30 wt% SiCnw@MXene9:1) can be found in Figs. S13 and S14.

On the other hand, the SiCnw@MXene9:1 layered foam/film PVDF nanocomposite (45% VF) exhibited a smaller peak SE_R_ of 0.015 dB and a broader optimal low reflection bandwidth of 3.2 dB at a foam thickness of 1.5 mm, as compared to those of the SiCnw@MXene7:1 layered foam/film PVDF nanocomposite (45% VF). With higher MXene content, the SiCnw@MXene7:1 layered foam/film PVDF nanocomposites exhibit higher impedance mismatch and reflection at 45% VF compared to those of SiCnw@MXene9:1 layered foam/film PVDF nanocomposite. This indicates that a higher VF is required to achieve optimal impedance matching of the absorber with higher MXene content.

As shown in Table S1, the foamability of PVDF/30 wt% SiCnw@MXene5:1 nanocomposite is significantly restricted due to the addition of MXene. A maximum VF of about 35% was obtained, which is insufficient to tune the impedance matching of the absorption phase (Fig. S9d).

Figure S15 illustrated some of the recent advances made in the development of EMI shielding materials [16, 30, 40, 46, 79–84], which are compared with the overall EMI shielding effectiveness and reflection of the layered foam/film PVDF nanocomposites reported in this study. The superior electrical conductivity is often prerequisite for achieving high EMI SE in the traditional EMI shielding materials. However, high electrical conductivity will also lead to a severe impedance mismatch and high reflectivity. As shown in Fig. S14, compared with other EMI shielding composites, the layered foam/film PVDF nanocomposite (30 wt% SiCnw@MXene 7:1, 65% VF) can achieve the average reflectivity (R) as low as 0.08 while maintaining efficient shielding effectiveness of 32.48 dB highlighting the supremacy of this work.

**Fig. 7:**
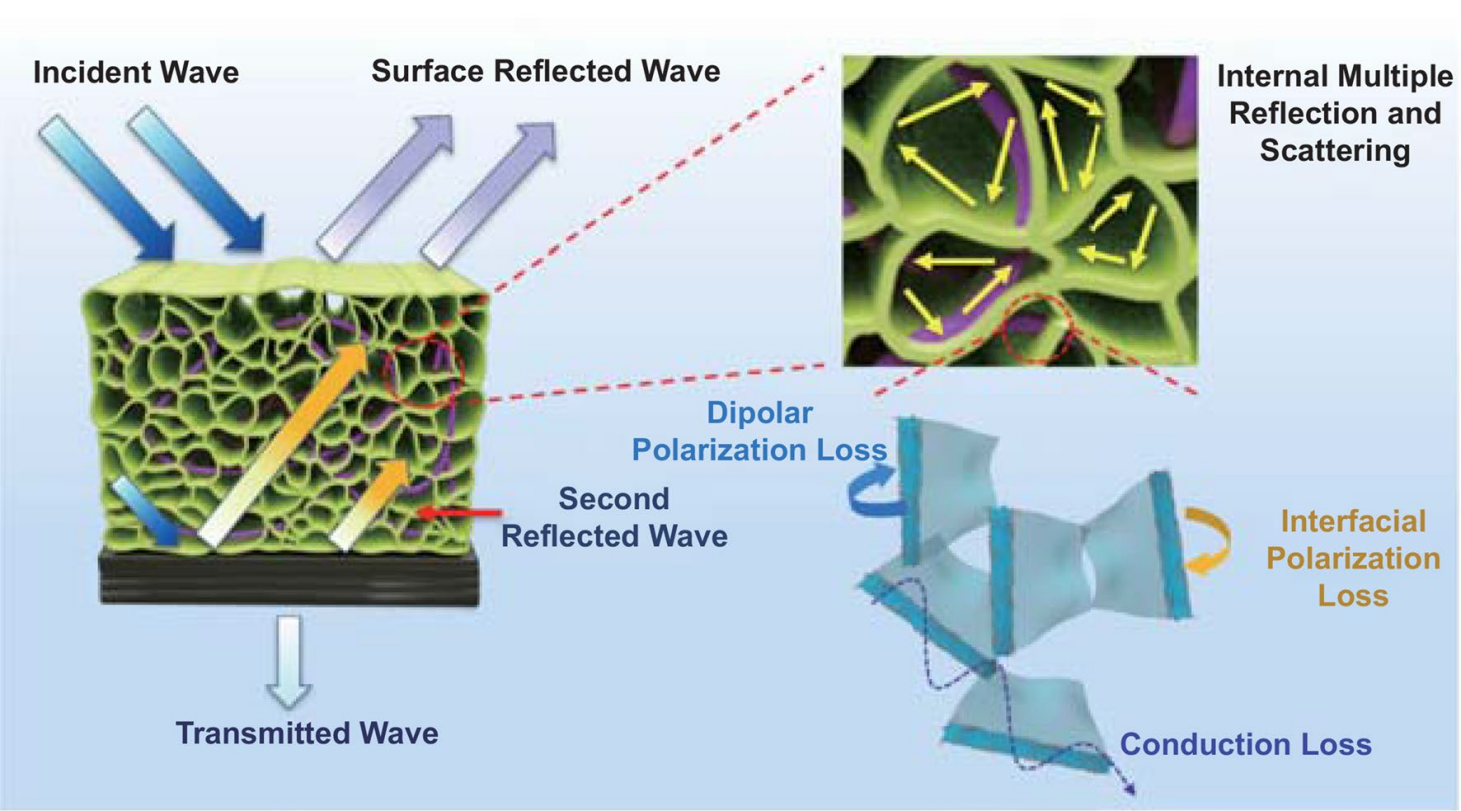
Schematic illustration of EM wave dissipation in the layered foam/film PVDF nanocomposites

The effective EMI shielding with ultra-low reflection efficiency of layered foam/film PVDF nanocomposites developed in this study can be attributed to the dissipation mechanisms presented in Fig. 7. The EM waves can penetrate the absorption layer and its microcellular structure because of good impedance matching. The penetrated waves will be dissipated by the numerous heterogeneous interfaces between SiCnw, MXene nanosheets and PVDF matrix via interfacial polarization loss, dipolar polarization loss and conduction loss. Furthermore, abundant solid–air interfaces obtained from the microcellular structure can trap the penetrated EM wave via scattering and multiple reflections. This prolongs the trajectory of the EM wave propagation within foams leading to further attenuation. The remaining propagating EM waves will be mostly reflected by the highly conductive reflection layer of PVDF/MWCNT/GnPs composite film and propagate through the absorption layer again. Finally, EM wave energy will be consumed by the interference between the second reflected wave and surface reflected EM wave, based on the law of quarter wavelength cancellation. In general, the layered foam/film PVDF nanocomposites achieved both efficient EMI SE and low reflectivity through (i) enhanced surface impedance matching of the foamed upper absorption phase, (ii) excellent EM wave attenuation via enhanced dielectric loss offered by the embedded hybrid heterostructures of SiCnw@MXene in the absorption layer, (iii) the efficient shielding effectiveness of the reflection layer achieved as a result of superior electrical conductivity and high reflection capability, and (iv) triggering quarter wavelength cancellation of incident EM wave by tuning the thickness of absorption phase.

## Summary and Conclusions

Layered foam/film PVDF nanocomposites with high-efficiency EMI SE and ultra-low reflection characteristics are introduced. The layered nanocomposites were composed of PVDF/SiCnw@MXene composite foam as the upper absorption layer and PVDF/MWCNT/GnPs composite film as the bottom reflection layer. The layered foam/film structure was developed by leveraging the different crystal melting temperatures of two grades of PVDF in the batch foaming process. The absorption layer's impedance matching and dissipation capability were effectively tuned by developing heterogeneous interfaces between SiCnw, MXene nanosheets and PVDF matrix, and by introducing microcellular structure. Meanwhile, the 0.4-mm-thick highly conductive PVDF/10 wt% MWCNT/10 wt% GnPs composite film performed as an excellent reflection layer. The synergy between absorption and reflection layers, along with optimized filler content/ratio and VF of the EM wave shielding and attenuation properties of the nanocomposites, was maximized. For instance, the layered foam/film PVDF nanocomposite (30 wt% SiCnw@MXene 7:1, 65% VF) exhibited an average EMI SE of 32.6 dB with low reflection bandwidth of 4 GHz and minimal SE_R_ of 3.1 × 10^–4^ dB over the Ku-band (12.4–18 GHz). The design of layered foam/film structure in this work presents a novel and simple strategy for developing high-efficiency, lightweight and wideband low reflection EMI shielding materials for cutting edge applications.

## Supporting Information

The SEM images of fabricated composites, XPS results, AC electrical conductivity and EMI shielding properties.

## Supplementary Information

Below is the link to the electronic supplementary material.

Supplementary file1 (PDF 1924 KB)
