# Supplementary material for: Layered Foam/Film Polymer Nanocomposites with Highly Efficient EMI Shielding Properties and Ultralow Reflection
Source: Nanomicro Lett. 2021 Dec 7;14:19. doi: 10.1007/s40820-021-00759-4 (PMC8651911; doi:10.1007/s40820-021-00759-4)
Supplement: Supplementary file 1 — Supplementary file1 (PDF 1924 KB) [file 40820_2021_759_MOESM1_ESM.pdf]

Supporting Information for

Layered Foam/Film Polymer Nanocomposites with Highly Efficient EMI Shielding Properties and Ultralow Reflection

Li Ma[1], Mahdi Hamidinejad[1, 2], Biao Zhao[1, 3, 4,] *, Caiyun Liang[1, 5], and Chul B. Park[1,] *

[1] Department of Mechanical and Industrial Engineering, University of Toronto, 5 King's College Road, Toronto, Ontario M5S 3G8, Canada

[2] Institute for Manufacturing, Department of Engineering, University of Cambridge, Cambridge CB3 0FS, United Kingdom

[3] Laboratory of Advanced Materials, Department of Materials Science, Collaborative Innovation Center of Chemistry for Energy Materials, Fudan University, Shanghai 200438, P. R. China

[4] Henan Key Laboratory of Aeronautical Materials and Application Technology, School of Material Science and Engineering, Zhengzhou University of Aeronautics, Zhengzhou, Henan 450046, P. R. China

[5] CAS Key Laboratory of High-Performance Synthetic Rubber and Its Composite Materials, Changchun Institute of Applied Chemistry, Chinese Academy of Sciences, Changchun 130022, P. R. China

* Corresponding authors. E-mail: park@mie.utoronto.ca (Chul B. Park), Biao_zhao@zua.edu.cn (Biao Zhao)

S1 Preparation of $Ti_3C_2T_x$ MXene Nanosheets

Firstly, 8 g of LiF powder was dissolved into100 ml of hydrochloric acid (9 N) under continuous stirring at 40 °C. Then, 5 g of $Ti_3C_2T_x$ powder was gradually added to the LiF/HCl solution under continuous stirring. After 24 h of stirring at 40 °C, the resulting acidic mixture was washed with DI water via centrifuge (4,000 rpm for 5 mins) for several cycles until the solution pH value was above 6. Then, the sediment of $Ti_3C_2T_x$ MXene in DI water was sonicated for 5 min and centrifuged at 3,500 rpm for 5 min. Finally, the dark green colloidal suspension containing few-layered $Ti_3C_2T_x$ nanosheets was carefully collected [S1]. And the colloidal suspension was then vacuum filtered into films and stored for the later preparation of PVDF-HFP/SiCnw/MXene ($Ti_3C_2T_x$) composites.

S2 Preparation of PDDA Modified SiCnw

Firstly, 1 g of SiCnw was gradually added to 250 mL of 0.4 wt% PDDA solution under continuous stirring for 12 h. Afterwards, the modified SiCnw was then collected using centrifuge and washed with DI water for three times. Finally, the modified SiCnw with

positive charge was dried in a vacuum oven at 60 °C overnight.

S3 Material Selection

PVDF is selected as matrix material in this work owing to its excellent chemical stability, UV-retardation, wear abrasion, hydrophobicity, and electroactive properties [S2-S5]. Also its outstanding dielectric properties can benefit the attenuation of EM waves by providing dipolar polarization loss and interfacial polarization loss [S6, S7].

S4 Foaming Mechanisms

In the batch foaming process, the microcellular structure is achieved by the following steps: (i) load the polymer matrix and saturate the chamber with a physical blowing agent, such as ScCO_2, at the set points of gas pressure and temperature (typically the set point of temperature is above the crystal melting temperature of semi-crystalline polymer), (ii) allow the CO_2 molecules dissolve into the polymer matrix by maintaining the temperature and saturating pressure, (iii) release the pressurized CO_2 gas, and cell nucleation occurs immediately due to significant thermodynamic instability within the polymer and gas system [S8, S9], (iv) nucleated cells start growing within the viscoelastic polymer melt, (v) and the final microcellular structure will be stabilized by solidifying the polymer matrix via rapid temperature drop in an ice bath.

S5 Supplementary Table and Figures

Table S1 Saturation temperature and void fraction of PVDF-HFP/SiCnw@MXene foams with different filler compositions

Void Fraction	T°C							
30wt% SiCnw: MXene	128°C	132°C	134°C	136°C	138°C	140°C	142°C	143°C
1:0	34.7%	45.2%	57.2%	65.0%	85.4%	-	-	-
9:1	-	34.3%	46.4%	54.6%	64.5%	-	-	-
7:1	-	-	38.1%	44.3%	54.2%	63.8%	-	-
5:1	-	-	-	-	-	24.2%	34.1%	30.0%

The void fraction of the foam layer was determined by using the average density of foam-film composites subtracting the density of the film layer times the ratio of layer thickness.

$$VF = \frac{\rho_{foamfilm} - \rho_{PVDF} \times \frac{t_{PVDF}}{t_{foamfilm}}}{\rho_{PVDF-HFP}} \quad (S1)$$

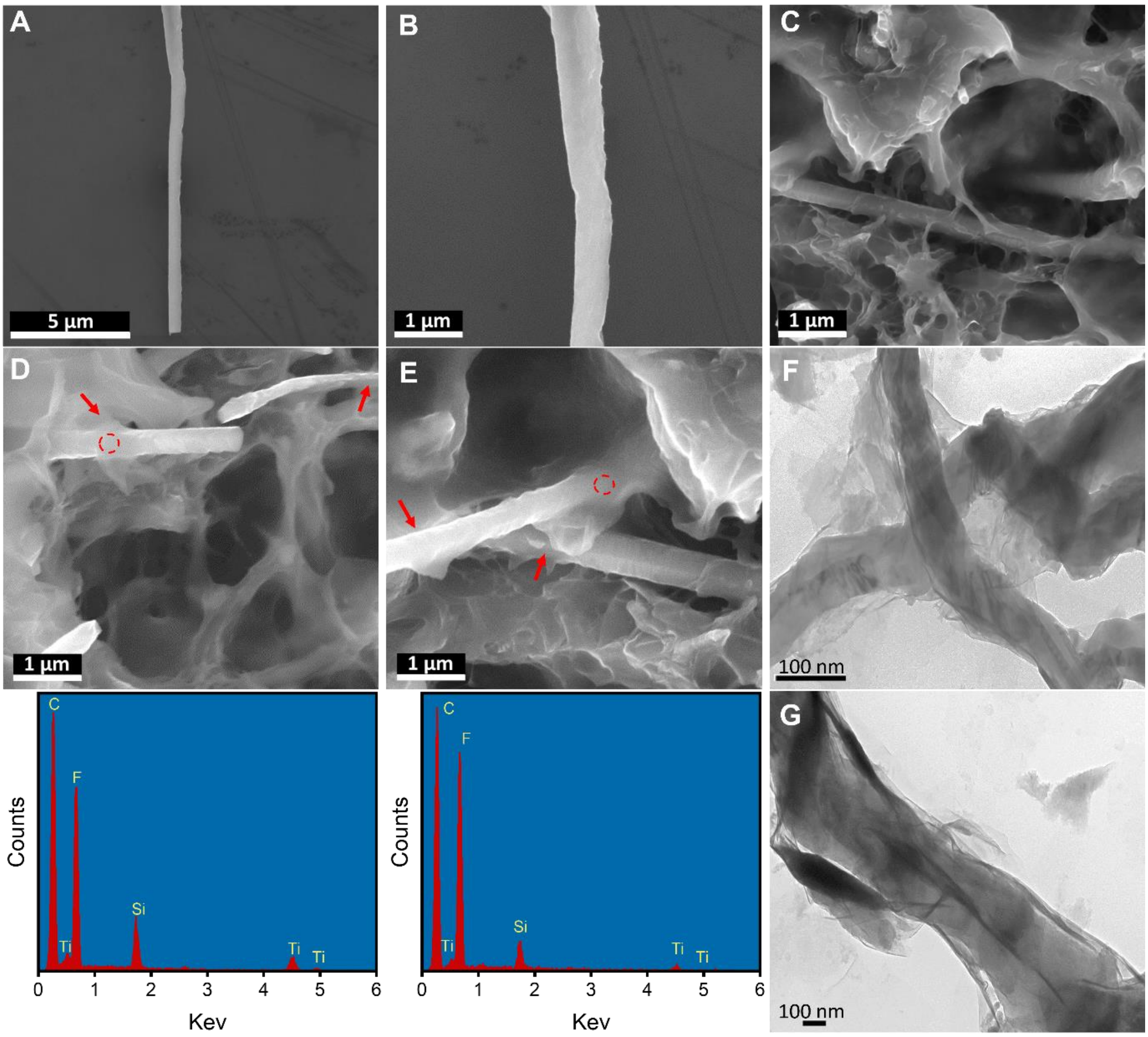

Fig. S1 (**A-B**) SEM images of SiCnw; (**C**) PVDF-HFP/SiCnw@MXene composite foam (30 wt%, 45% VF); (**D-E**) SEM image of EDX-SEM images of PVDF-HFP/SiCnw@MXene composite foam (30 wt%, 45% VF); and (F-G) TEM images of SiCnw@f- $Ti_3C_2T_x$

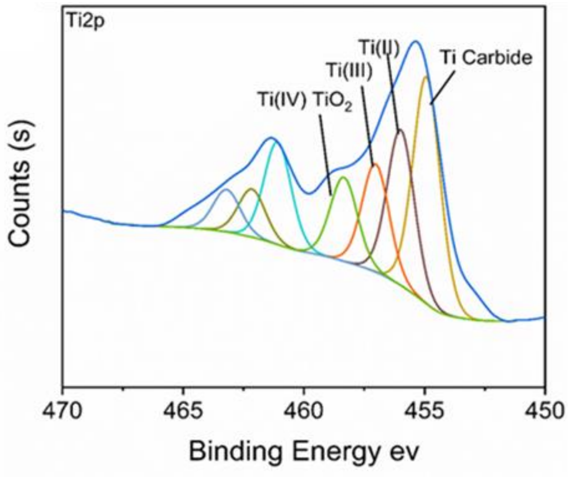

Fig. S2 XPS result of f- $Ti_3C_2T_X$ MXene nanosheets

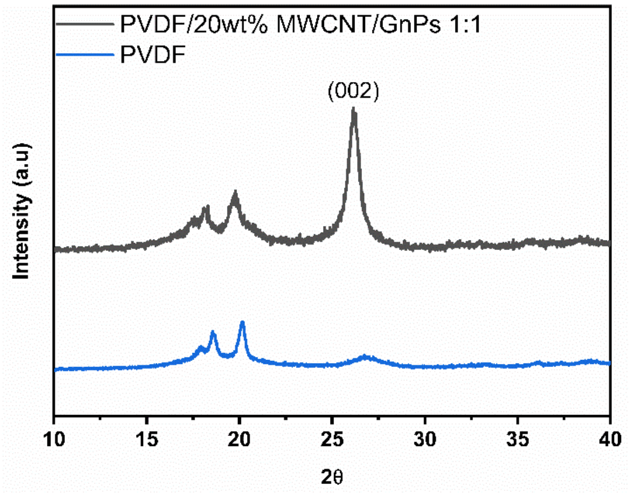

Fig. S3 XRD patterns for PVDF nanocomposite

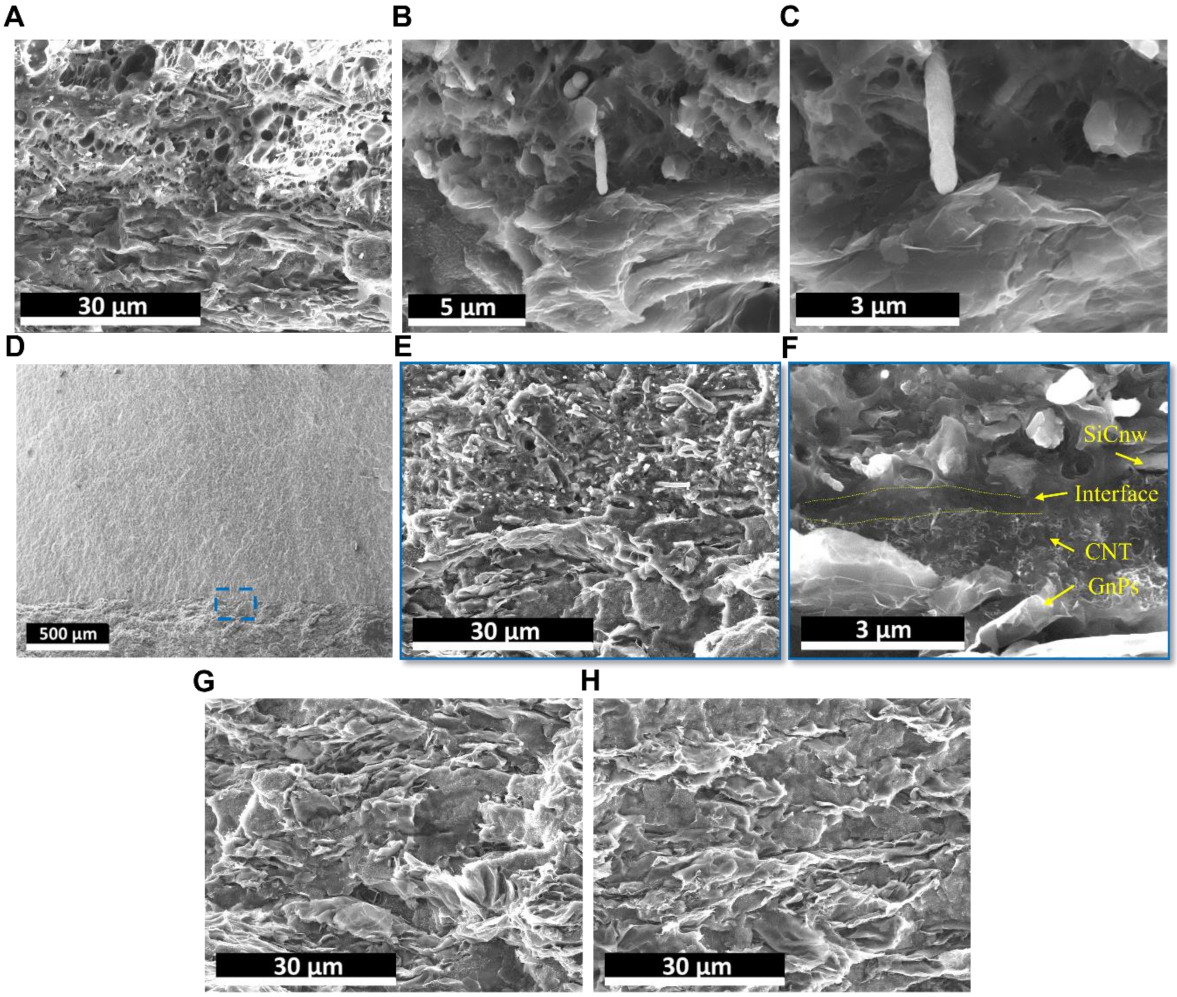

Fig. S4 (**A-C**) SEM images of interface area for the layered foam/film PVDF nanocomposite (30% SiCnw@MXene7:1, VF:45%); (D-F) SEM images of interface area for the layered PVDF nanocomposite (30% SiCnw@MXene7:1, solid); (**G**) SEM images for bottom reflection phase (20% MWCNT: GnPs= 1:1) before foaming; and (**H**) SEM images for bottom reflection phase after foaming

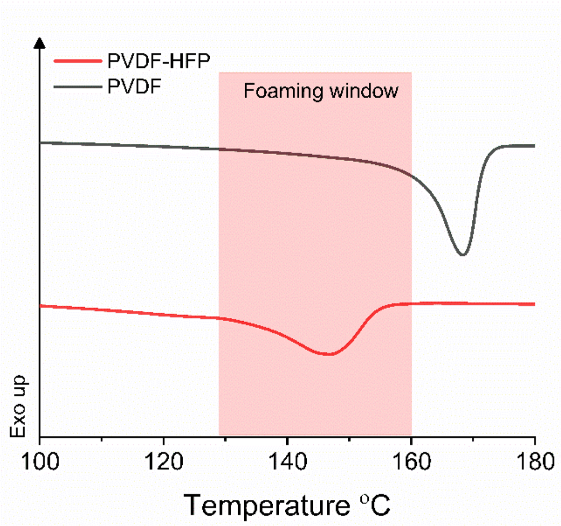

Fig. S5 Endothermal plot of 10 °C/min heating curve for Kynar-2800 and Kynar-740 matrix

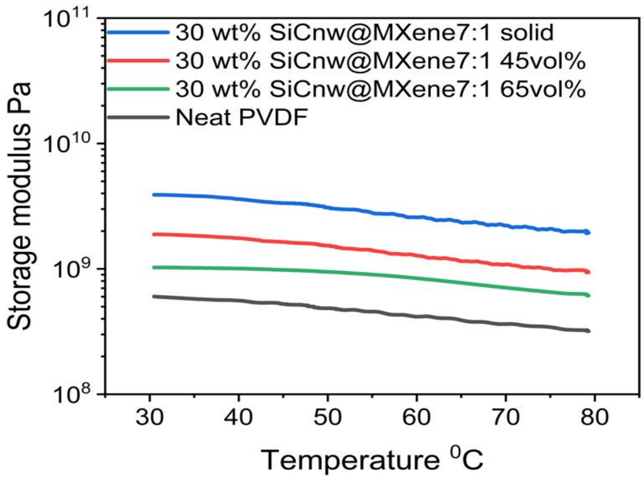

Fig. S6 The storage modulus for 1.5 mm of layered foam/film PVDF nanocomposites (30 wt% SiCnw@MXene7:1) with various void fractions and neat PVDF

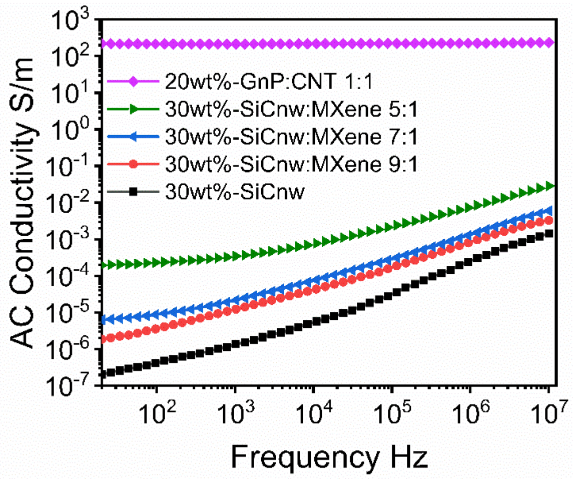

Fig. S7 The AC conductivity for individual PVDF nanocomposite with various filler compositions

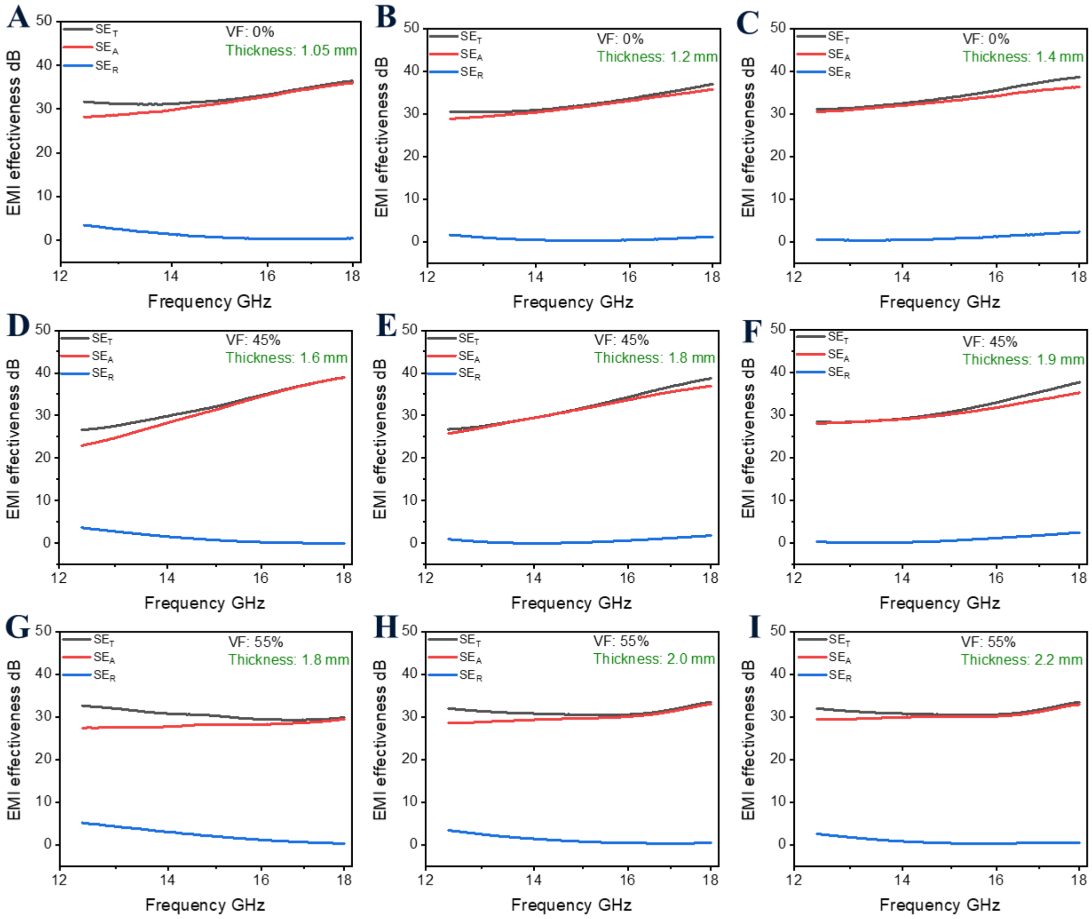

Fig. S8 EMI SE of the layered foam-film PVDF nanocomposites (30 wt% SiCnw) with the various void fraction and thicknesses of absorption phase. (**A-C**) 0% VF; (**D-F**) 45% VF; and (G-I) 55% VF

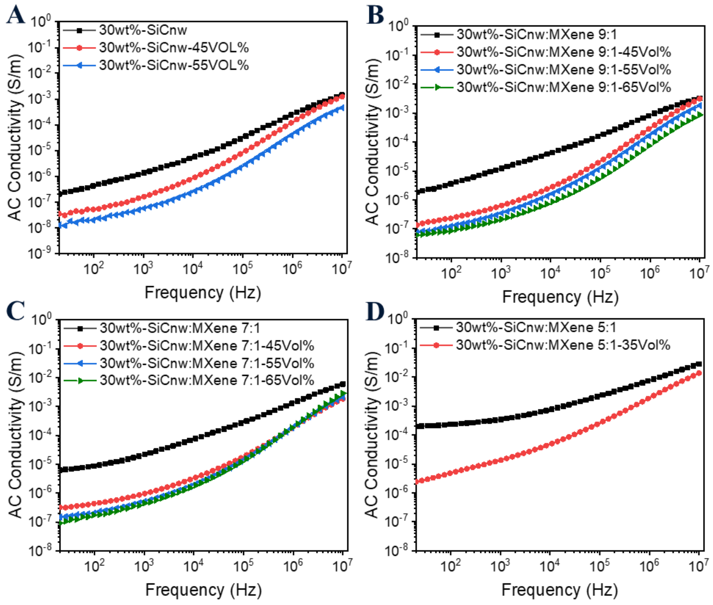

Fig. S9 AC conductivity of the foamed absorption phase with various filler compositions and void fraction

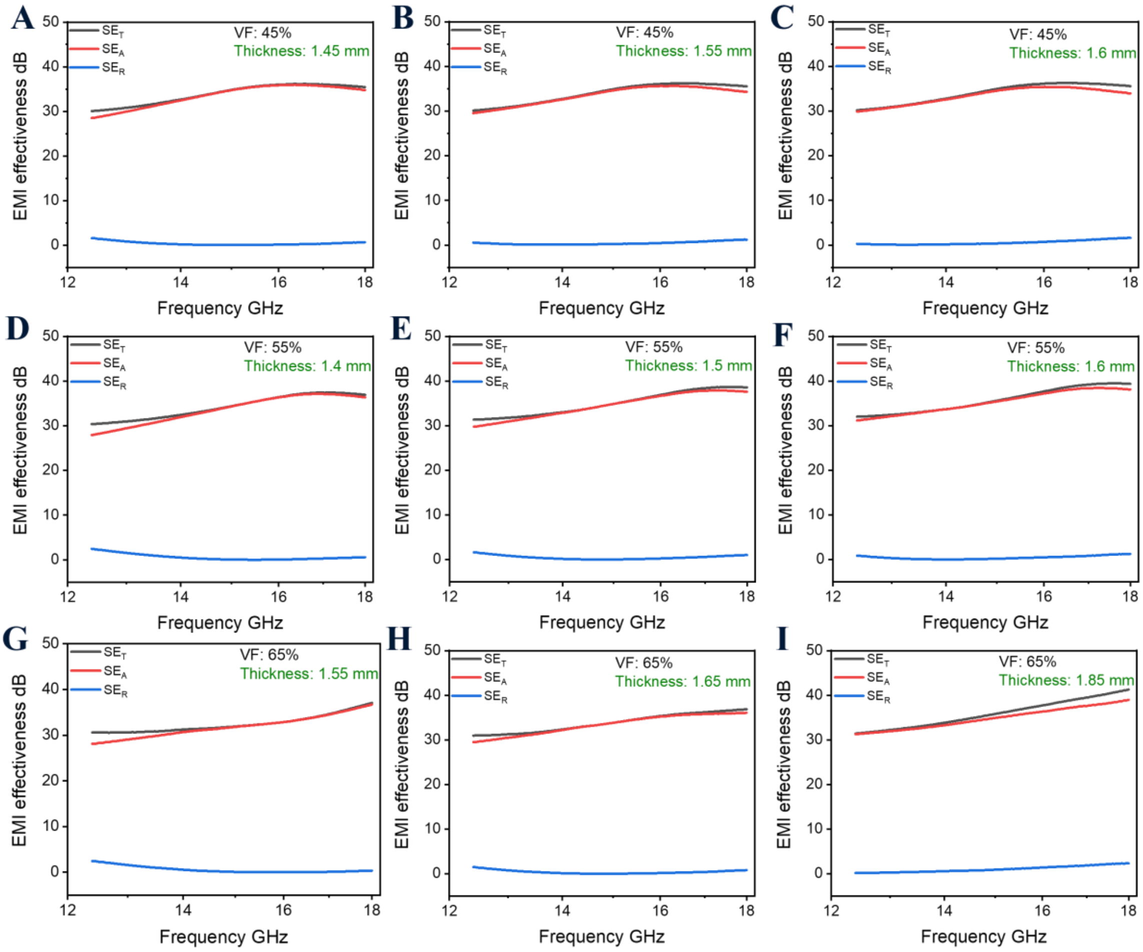

Fig. S10 EMI SE of the layered foam-film PVDF nanocomposites (30 wt% SiCnw@MXene7:1) with the various void fraction and thicknesses of absorption phase. (**A-C**) 45% VF; (**D-F**) 55% VF; and (**G-I**) 65% VF

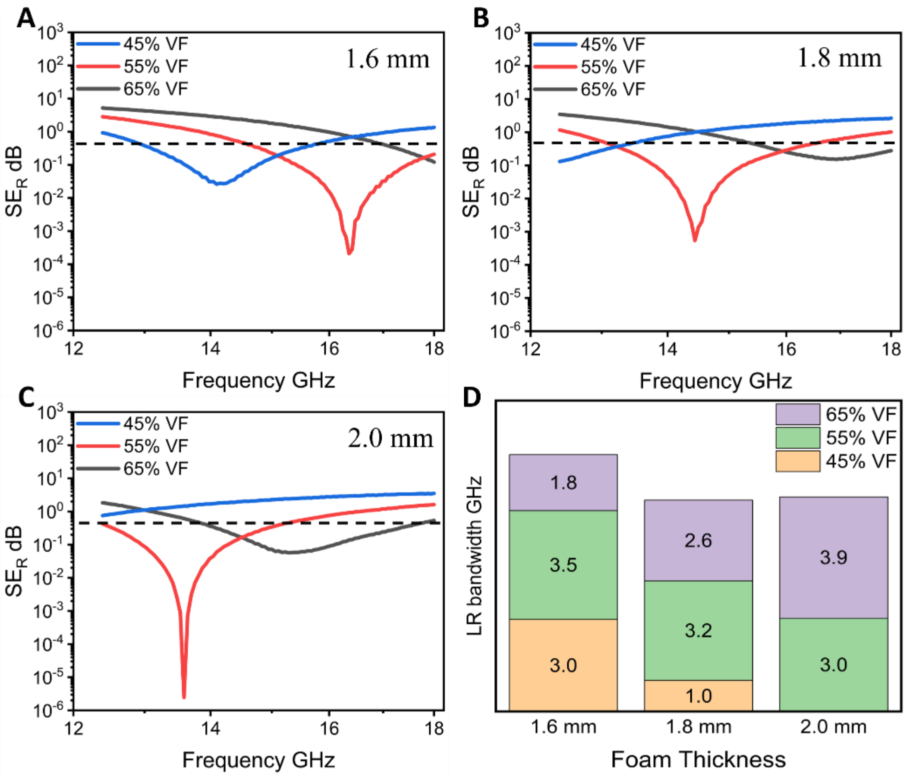

Fig. S11 EMI reflection performance for layered foam/film PVDF nanocomposites (30 wt% SiCnw@MXene9:1) corresponds to various void fractions of absorption phase at the same thickness; (**A**) SE_R at 1.6 mm; (**B**) SE_R at 1.8 mm; (**C**) SE_R at 2.0 mm; and (**D**) low-reflection bandwidth

As shown in Fig. S11A-C, at the same foam thickness, the peak value of SE_R shifts towards higher frequency with an increasing void fraction of the absorption layer. According to the law of quarter wavelength cancelation, the shifted SE_R values are attributed to the decreased complex permittivity. This can be further supported in Fig. S12F, the complex permittivity of layered composites decreased with the increase of void fraction, and all complex permittivity exhibited the frequency-dependent behavior. Therefore, the optimal thickness for each VF is not constant. By increasing the void fraction from 55% to 65%, the layered foam/film PVDF nanocomposite (9:1, 65% VF) obtained a much higher minimum SE_R value, increased from $2.48 \times 10^{-6}$to 5.41×10^{-2} dB. This is attributed to the excessively high void content, which resulted in insufficient permittivity and dissipation capability. This can be further supported in Fig. S12A, where the SE_A of the individual layer of PVDF nanocomposite foam (30 wt% SiCnw@MXene 9:1, 1.8 mm) decreased from 3.75 to 3.12 dB as the void fraction increased from 55% to 65%.

In Fig. S11D, among all three chosen thicknesses (1.6, 1.8, and 2.0 mm), layered foam/film PVDF nanocomposite with higher void fraction exhibited the broader optimal low reflection (LR) bandwidth, due to the enhanced impedance matching. And the layered foam/film PVDF nanocomposite (9:1, 65% VF) obtained the broadest LR bandwidth of 3.9 GHz with a thickness of 2.0 mm.

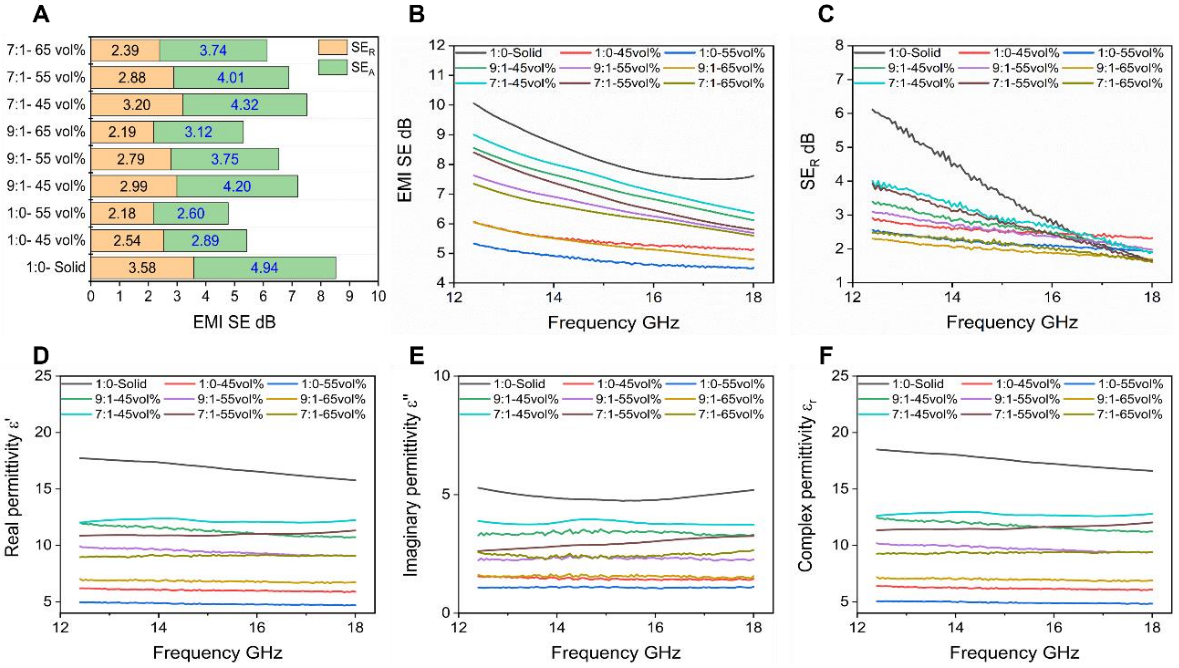

Fig. S12 EMI Shielding properties for PVDF nanocomposite foams (30 wt% filler concentration, 1.8 mm thickness) with various SiCnw: MXene ratios (1:0, 9:1 and 7:1) and void fraction (45% VF, 55% VF and 65% VF), (**A**) average SE_R, SE_A and SE_T; (**B**) broadband EMI SE; (**C**) broadband SE_R; (**D**) broadband real permittivity ε'; (**E**) broadband imaginary permittivity ε''; and (**F**) broadband complex permittivity ε_r

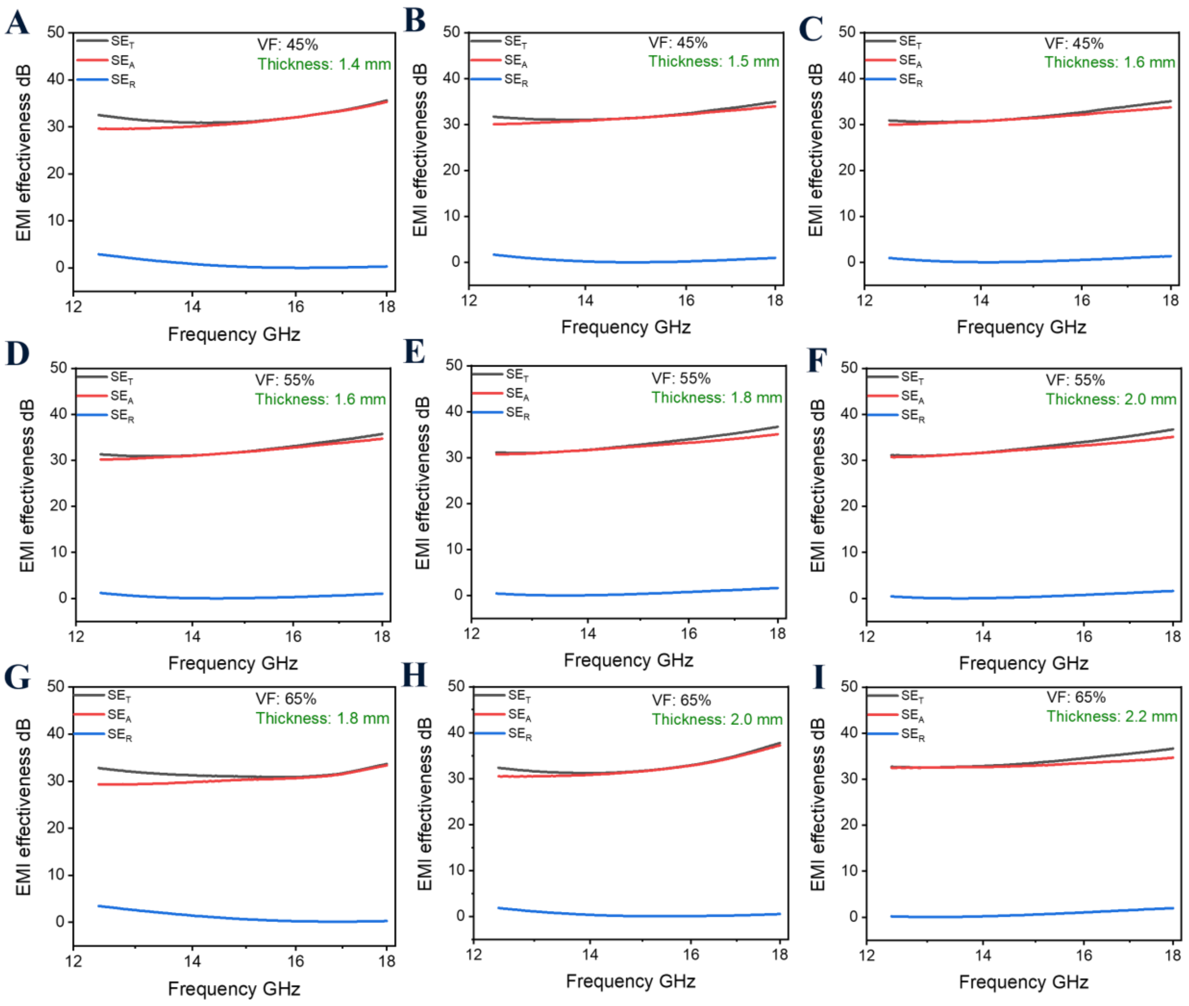

Fig. S13 The EMI SE of the layered foam-film PVDF nanocomposites (30 wt% SiCnw@MXene9:1) with the various void fraction and thicknesses of absorption phase. (**A-C**) 45% VF; (**D-F**) 55% VF; and (**G-I**) 65% VF

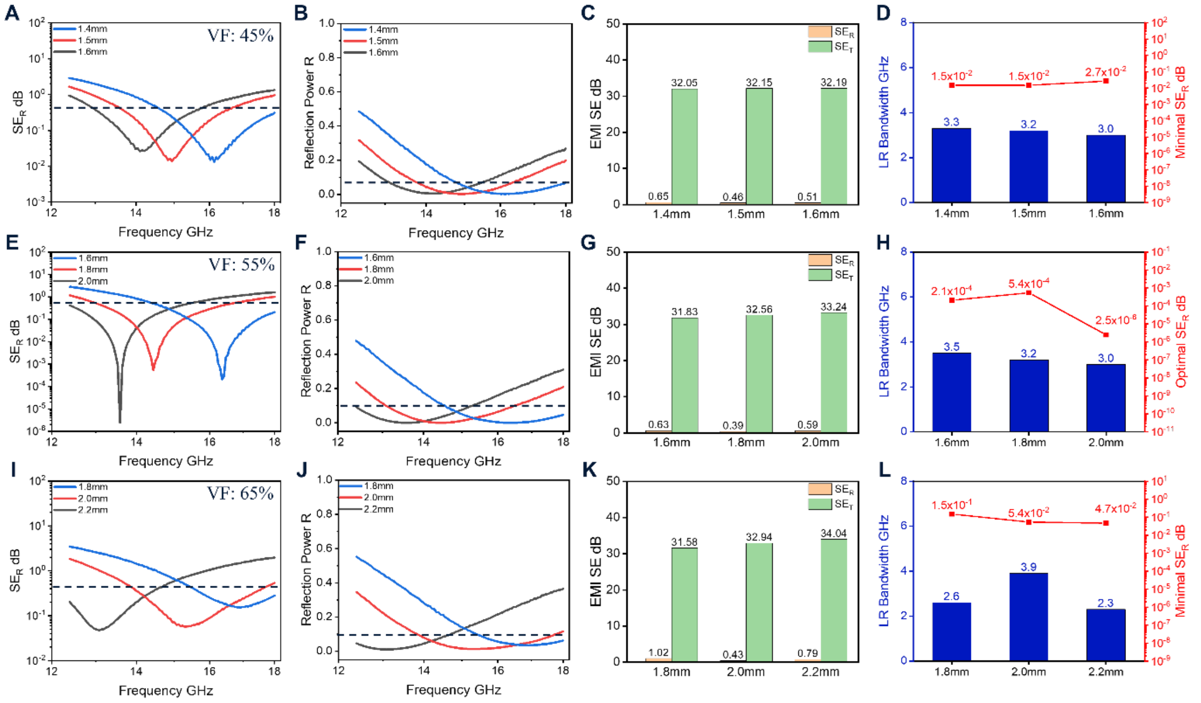

Fig. S14 EMI shielding performance for layered foam-film PVDF nanocomposites (30 wt% SiCnw@MXene9:1) corresponds to different matching thicknesses of absorption phase; (**A**) SE_R; (**B**) reflection power R; (**C**) average value of SE_R and SE_T; and (**D**) low-reflection bandwidth and minimal SE_R value. Dependence of void fraction: (**A-D**) 45% VF; (**E-H**) 55% VF; and (**I-L**) 65% VF

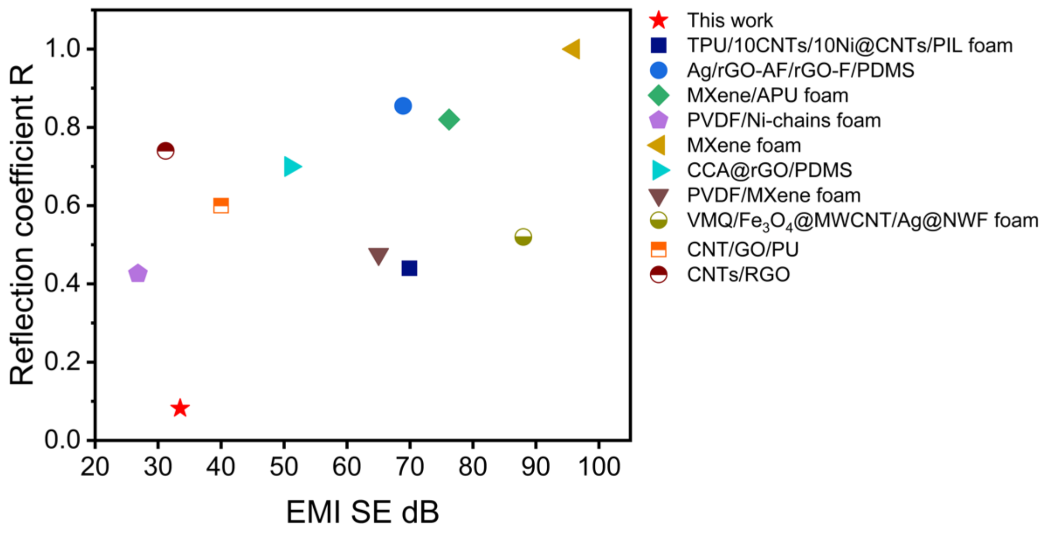

Fig. S15 Comparison of EMI SE and the R value of this work with other EMI shielding composites

Supplementary References

[S1] M. Naguib, O. Mashtalir, J. Carle, V. Presser, Jun Lu et al., Two-dimensional transition metal carbides. ACS Nano **6**, 1322-1331 (2012). https://doi.org/10.1021/nn204153h

[S2] F. Chen, Y. Lu, X. Liu, J. Song, G. He et al., Table salt as a template to prepare reusable porous PVDF-MWCNT foam for separation of immiscible oils/organic solvents and corrosive aqueous solutions. Adv. Funct. Mater. **27**, 41 (2017). https://doi.org/10.1002/adfm.201702926

[S3] C.Y. Kuo, H.N. Lin, H.A. Tsai, D.M. Wang, J.Y. Lai, Fabrication of a high hydrophobic PVDF membrane via nonsolvent induced phase separation. Desalination **233**, 40-47 (2008). https://doi.org/10.1016/j.desal.2007.09.025

[S4] P. Martins, A. C. Lopes, S. Lanceros-Mendez, Electroactive phases of poly(vinylidene fluoride): Determination, processing and applications. Prog. Polym. Sci. **39**, 683-706 (2014). https://doi.org/10.1016/j.progpolymsci.2013.07.006

[S5] H. Wang, Z. Liu, E. Wang, R. Yuan, D. Gao et al., A robust superhydrophobic PVDF composite coating with wear/corrosion-resistance properties. Appl. Surf. Sci. **332**, 518-524 (2015). https://doi.org/10.1016/j.apsusc.2015.01.213

[S6] K. Rajavel, S. Luo, Y. Wan, X. Yua, Y. Hu et al., 2D $Ti_3C_2T_x$ MXene/polyvinylidene fluoride (PVDF) nanocomposites for attenuation of electromagnetic radiation with excellent heat dissipation. Compos. Part A Appl. Sci. Manuf. **129**, 105693 (2019). https://doi.org/10.1016/j.compositesa.2019.105693

[S7] R. Li, L. Ding, Q. Gao, H. Zhang, D. Zeng et al., Tuning of anisotropic electrical conductivity and enhancement of EMI shielding of polymer composite foam via CO_2-assisted delamination and orientation of MXene. Chem. Eng. J. **415**, 128930 (2021). https://doi.org/10.1016/j.cej.2021.128930

[S8] S.N. Leung, C.B. Park, D. Xu, H. Li, R.G. Fenton, Computer simulation of bubble-growth phenomena in foaming. Ind. Eng. Chem. Res. **45**, 23, 7823–7831 (2006). https://doi.org/10.1021/ie060295a

[S9] S.N. Leung, A. Wong, Q. Guo, C.B. Park, J.H. Zong, Change in the critical nucleation radius and its impact on cell stability during polymeric foaming processes. Chem. Eng. Sci., **64**, 23, 4899-4907 (2009). https://doi.org/10.1016/j.ces.2009.07.025
